# Prefrontal Disinhibition in Social Fear: A Vital Action of Somatostatin Interneurons

**DOI:** 10.3389/fncel.2020.611732

**Published:** 2020-12-17

**Authors:** Jun Wang, Yuanyuan Tian, Ling-Hui Zeng, Han Xu

**Affiliations:** ^1^Department of Neurobiology and Department of Neurology of the Second Affiliated Hospital, Zhejiang University School of Medicine, Hangzhou, China; ^2^NHC and CAMS Key Laboratory of Medical Neurobiology, MOE Frontier Science Center for Brain Research and Brain-Machine Integration, School of Brain Science and Brain Medicine, Zhejiang University, Hangzhou, China; ^3^Department of Pharmacology, School of Medicine, Zhejiang University City College, Hangzhou, China

**Keywords:** social anxiety disorder, social fear, interneuron, disinhibition, prefrontal cortex

## Abstract

Social fear and avoidance of social partners and social situations represent the core behavioral symptom of Social Anxiety Disorder (SAD), a prevalent psychiatric disorder worldwide. The pathological mechanism of SAD remains elusive and there are no specific and satisfactory therapeutic options currently available. With the development of appropriate animal models, growing studies start to unravel neuronal circuit mechanisms underlying social fear, and underscore a fundamental role of the prefrontal cortex (PFC). Prefrontal cortical functions are implemented by a finely wired microcircuit composed of excitatory principal neurons (PNs) and diverse subtypes of inhibitory interneurons (INs). Disinhibition, defined as a break in inhibition *via* interactions between IN subtypes that enhances the output of excitatory PNs, has recently been discovered to serve as an efficient strategy in cortical information processing. Here, we review the rodent animal models of social fear, the prefrontal IN diversity, and their circuits with a particular emphasis on a novel disinhibitory microcircuit mediated by somatostatin-expressing INs in gating social fear behavior. The INs subtype distinct and microcircuit-based mechanism advances our understanding of the etiology of social fear and sheds light on developing future treatment of neuropsychiatric disorders associated with social fear.

## Introduction

The fear response to an imminent threat is an adaptive behavior and is essential to avoid danger in the environment for animals and humans. However, persistent and unnecessary fear response represents a maladaptive behavior evident in a large number of psychiatric diseases such as posttraumatic stress disorder (PTSD) and anxiety disorders (Buff et al., 2016; Nees et al., 2018). Intense and persistent fear and avoidance of social situations represent the core behavioral symptom of social anxiety disorder (SAD), a prevalent psychiatric disorder worldwide (Kessler et al., 2005a,b; Stein and Stein, 2008; Leichsenring and Leweke, 2017). Social fear makes even the simplest daily task challenging and distressful and literally disconnects individuals afflicted from others and society. Unfortunately, there are no satisfactory therapeutic options currently available (Stein and Stein, 2008; Dos Santos et al., 2019). The pathological mechanism underlying SAD is undetermined partly due to a lack of specific animal models (Toth et al., 2012, 2013; Toth and Neumann, 2013). Recently, thanks to the effort of several groups of researchers, a couple of experimental paradigms have been developed to induce social fear in rodents. Importantly, these paradigms reliably induce robust behavioral changes recapitulating core behavioral symptoms of SAD in humans, without affecting non-social behaviors such as locomotion, general anxiety, and depressive-like behaviors (Toth et al., 2012; Toth and Neumann, 2013; Franklin et al., 2017; Xu et al., 2019). Notably, by using these rodent animal models, neuroscientists start to dissect the neuronal circuit substrates underlying social fear (Franklin et al., 2017; Xu et al., 2019).

Accumulating evidence from human functional brain imaging studies suggests that the prefrontal cortex (PFC) contributes essentially to the processes of social fear responses (Buff et al., 2016; Kawashima et al., 2016). The PFC is composed of a majority of principal neurons (PNs) and a minority of inhibitory interneurons (INs) which exhibit remarkable diversity in morphology, physiological properties, immunohistochemical characteristics, and connectivity (Kawaguchi and Kubota, 1997; Rudy et al., 2011; Xu et al., 2013; Hattori et al., 2017). Different subtypes of INs could effectively control cortical network activity *via* feedforward, feedback inhibition, and disinhibition (Xu et al., 2013, 2019; Tremblay et al., 2016). Both human and animal studies found that an exquisite balance between excitation and inhibition plays a fundamental role in cortical functions (Rubenstein and Merzenich, 2003; Yizhar et al., 2011; Sohal and Rubenstein, 2019). Moreover, the exact activity patterns of specific prefrontal IN subtypes and their precise microcircuit mechanism in fear-related behaviors including social fear start to emerge (Xu et al., 2019; Cummings and Clem, 2020).

In this review, we will first briefly introduce SAD and summarize major attempts in developing proper experimental paradigms to induce social fear in rodents. Then, we will discuss studies in the exploration of pathological mechanisms underlying social fear by using these rodent animal models. Given the complexity and multidimensional nature of social fear behavior, we will focus our discussion on the brain region of PFC. In specific, we will discuss prefrontal IN diversity and their microcircuits in the context of social fear regulation. Particularly, we will highlight a newly discovered disinhibitory microcircuit in the PFC *via* interactions between subtypes of INs. We suggest that prefrontal disinhibition mediated by somatostatin-expressing (SST^+^) INs represents an essential circuit mechanism in the regulation of social fear behavior.

## Social Fear and Animal Models of Social Fear

SAD, also known as social phobia, is one of the most frequent psychiatric illness, with a worldwide lifetime prevalence of up to 13% (Kessler et al., 2005a,b; Stein and Stein, 2008; Leichsenring and Leweke, 2017), and is more prevalent in adolescents than in adults (Stein and Stein, 2008; Leichsenring and Leweke, 2017). SAD is essentially characterized by persistent avoidance, anxiety, or fear of social or performance situations (Stein and Stein, 2008; Leichsenring and Leweke, 2017). Indeed, social fear and social avoidance is the core behavioral symptom of SAD in clinical diagnosis. In addition to SAD, social fear or avoidance of social situations is also commonly observed in many other neuropsychiatric disorders, such as autism, schizophrenia, and depression (Jones et al., 2017). Social fear leads to low self-esteem and disconnects individuals from others and society to a varying degree and can cause devastating consequences on the individuals’ daily life. Besides, it also causes high social and medical costs to the families afflicted and the society (American Psychiatric Publishing, 2013). Current treatments involve psychotherapy, pharmacotherapy, or a combination of both. These treatment options are effective for some individuals suffering from the disorder. However, the problem is that only partial remission of symptoms can be achieved and the recurrence rate after discontinuation of treatments is high (Blanco et al., 2002). What is even worse, for up to 30–40% of patients, these exiting treatments do not work (Blanco et al., 2002; Leichsenring and Leweke, 2017). Essentially, a deeper understanding of the pathological mechanisms underlying SAD is urgently needed.

Animal models offer valuable tools for understanding the biological mechanisms involved and finding more effective therapeutic targets for many diseases. However, it has not been successful in developing appropriate animal models for SAD. It has been shown that social fear and social avoidance could be reliably induced with several experimental paradigms, including social conflict (Huhman, 2006), foot shock (Haller and Bakos, 2002), social isolation (Hermes et al., 2011), and maternal separation (Niwa et al., 2011; for review, Toth and Neumann, 2013). Unfortunately, none of these paradigms produced behavioral outcomes that are specified in the social domain. Instead, other phenotypical changes were also evident in these experimental animal models, such as alterations in general anxiety, locomotor functions, as well as depressive-like behaviors (Toth and Neumann, 2013). Therefore, more specific animal models with no confounding factors are required to probe the underlying substrates of social fear. Such animal models are also useful for screening drugs for psychiatric disorders associated with social fear.

**Figure 1:**
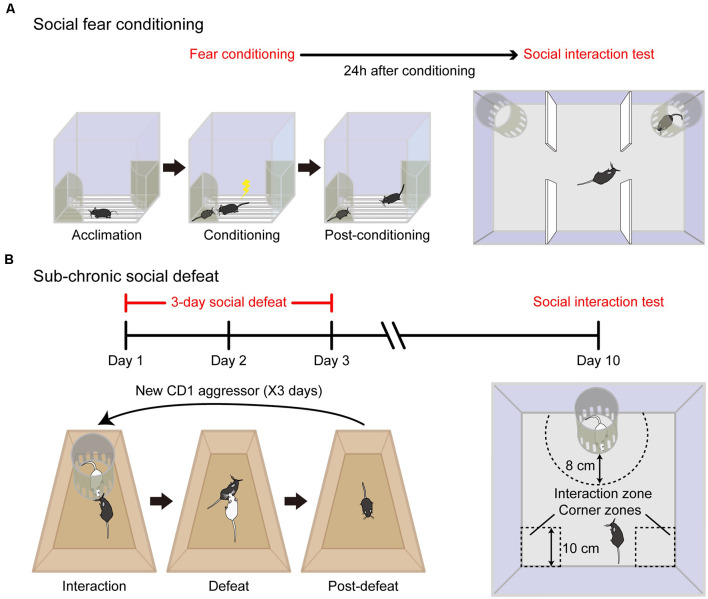
Specific animal models of social fear. **(A)** Schematic diagram of the social fear conditioning (SFC) paradigm. The experimental mouse was first allowed to acclimate to the conditioning chamber (Acclimation) and then a stimulus mouse was introduced to one of the stimulus cages placed on opposing corners of the conditioning chamber. During the conditioning session, the experimental mouse was allowed to freely interact with the stimulus mouse, while a foot shock was delivered each time when it approached and investigated the stimulus mouse (Conditioning). After conditioning, the procedure was extended to a longer duration to reinforce behavioral adaption (Post-conditioning). Adapted from Xu et al. (2019). **(B)** Schematic diagram of sub-chronic social defeat paradigm. For three consecutive days, an unfamiliar aggressive male CD1 intruder mouse (white color) was introduced to the home cage of singly-housed adult C57BL/6J male mice (black color). The intruder was confined within a Plexiglas stimulus cage (10 cm in diameter) for the first 5 min (interaction) and then was allowed to attack the experimental mouse for 10 min (defeat) and withdraw immediately after social confrontations (post-defeat). After 1 week recovery, the expression of social fear to an unfamiliar CD1 mouse was detected in an open field. Social avoidance was assessed by relative time spent in the interaction zone to corner zones.

To develop rodent models of social fear with more specificity, a couple of elegant studies have recently been conducted by making use of either social fear conditioning (SFC) or sub-chronic social defeat (Figure 1). The SFC paradigm was first introduced by Toth et al. (2012, 2013), which is based on the principle of operant fear conditioning by paring a conspecific social investigation with physical punishment (an electric foot shock). On the conditioning day, the experimental mouse was allowed to acclimate to the conditioning chamber with a floor consisting of a stainless-steel grid that delivers electric foot shocks and an empty wire mesh cage placed near a wall of the chamber. Then an unfamiliar conspecific mouse with matched gender was introduced to the wire mesh cage as a social stimulus. The experimental mouse was allowed to freely investigate the stimulus mouse before conditioning, and then an electric foot shock was manually delivered to the conditioned mouse each time when it approached and investigated the social stimulus mouse. After the conditioning, the conditioned mouse showed a dramatic reduction in the time of social investigation to unfamiliar conspecific mouse and other aversive responses toward the stimulus mouse. These behavioral changes reflect the successful induction of social fear in the conditioned mouse. In contrast to previous paradigms, the social fear mouse induced by the SFC paradigm showed no alterations in locomotion, general anxiety, or depressive-like behavior. Therefore, SFC is a reliable paradigm to induce social fear in mice with good specificity (Toth et al., 2012).

In a more recent study by us (Xu et al., 2019), we adopted the conditioning paradigm pioneered by Toth et al. (2012) and made several significant improvements. First, to ensure consistency of conditioning criteria and to reduce the behavioral variation among conditioned subjects, social contacts were monitored, and electric foot shocks were delivered automatically with a computerized conditioning unit equipped with a video tracking system. Second, to ensure fear acquisition specifically to the stimulus mouse but not to the cage, two identical cages were placed at each of two opposing corners of the conditioning unit with one containing a stimulus mouse and the other remaining empty during the conditioning procedure. Third, to reinforce behavioral adaptation of the conditioned mouse, the conditioning procedure was extended to a longer duration (20 min) although the experimental mice usually did not investigate stimulus mice and thus did not get foot shocks any longer after 5 min. Moreover, we employed C57BL/6J mice for the SFC paradigm instead of CD1 mice or rats by Toth et al. (2012, 2013) and therefore extended the application of this conditioning paradigm to a more broadly used species. This extension is important for future studies aimed to elucidate the etiology of social fear given that a large number of genetic resources and tools are readily available for C57BL/6J mouse lines. Consistent with the findings reported by Toth et al. (2012), the conditioned mice spent significantly less time with the stimulus mouse in a three-chamber social interaction test and exhibited significantly fewer approach times to social stimulus in a social preference-avoidance test. Besides, conditioned mice approached the stimulus mouse in a stretched posture and at a slow speed, two behavioral indicators of an elevated fear state in rodents that were absent in unconditioned control animals. Therefore, our modified conditioning paradigm is robust and reliable. Importantly, conditioned mice behaved normally in response to a novel object and exhibited no alterations in locomotion, general anxiety, and depressive-like behaviors, validating the specificity of behavioral changes in the social domain.

We also compared another social fear model that is induced by social defeat (Xu et al., 2019), which is adapted from a previous study conducted by Franklin et al. (2017). Unlike the SFC that relies on an artificial punishment (electric foot shock), social defeat happens in an experimental setting comparable to the mouse’s natural environment, that is, exposure to an aggressor. Repeated social defeat is widely employed as a standardized protocol to induce depressive-like behaviors in C57BL/6J mice (Golden et al., 2011). However, there are several significant differences in using social defeat to induce social fear. First, the duration of social defeat is shorter to establish a social fear mouse model than that for a depressive mouse model. The experimental C57BL/6J mice are exposed to agonistic social confrontations with an aggressive CD1 mouse for three consecutive days (Xu et al., 2019) for the social fear mouse model (so it is called sub-chronic social defeat) compared to a couple of weeks for depression (chronic social defeat). Second, to induce social fear an aggressive CD1 mouse is introduced into the home cage of experimental C57BL/6J mice and withdrawn immediately after social confrontations. In contrast, to induce depression the C57BL/6J mouse was living in a shared home cage with a CD1 mouse separated by a clear perforated divider (Golden et al., 2011). In this manner, the defeated mouse is subjected to continuous psychological stress from sensory interaction with the aggressor for the entire modeling period which facilitates its behavioral adaptions. As a consequence, after chronic social defeat, some mice exhibit specific depressive-like behaviors (termed “susceptible”) and the others have no change (termed “resilient”; Golden et al., 2011). In comparison, the mice subjected to sub-chronic social defeat showed a reduction in social investigations without alterations in locomotion, general anxiety, and depressive-like behaviors (Xu et al., 2019). Note that, for the SFC paradigm, the conditioned mouse develops a tight association between social stimulus and foot shock after SFC, and the animal shows social fear behavior to the stimulus mouse. Similarly, after sub-chronic defeat, the defeated mouse shows submission to the aggressive intruder. Despite the behavioral similarity of these two animal models, it is still an open question whether these defensive behaviors share the same neural circuits or not.

## Functions of the Prefrontal Cortex in Social Fear

Recent functional brain imaging studies have identified abnormal activities in several brain regions of patients with SAD (Zhu et al., 2017; Doruyter et al., 2018). These brain regions largely belong to the limbic system including the amygdala (Kraus et al., 2018; Figel et al., 2019; Frick et al., 2020), bed nucleus of the stria terminalis (BNST; Figel et al., 2019), and PFC (Buff et al., 2016; Kawashima et al., 2016; Frick et al., 2020). In particular, both near-infrared spectroscopy (Kawashima et al., 2016) and functional magnetic resonance imaging (fMRI) studies (Buff et al., 2016) revealed that hyperactivity of PFC is tightly linked to excessive and long-lasting fear states in patients with SAD. In human, the PFC is mainly composed of four subregions, namely, orbitofrontal cortex (OFC), dorsolateral PFC, ventrolateral PFC, and mPFC (Ko, 2017), which have an important role in the processing of complicated cognitive and executive behaviors (e.g., social behaviors; Amodio and Frith, 2006) as well as emotion (Etkin et al., 2011). Although it is still controversial, emerging evidence suggests that it is anatomically comparable and functional homologous between human and rodent PFC structures (Dalley et al., 2004). The PFC regions in rodents can be categorized into three major subregions: the dorsal part of the medial PFC (dmPFC), ventral part of the medial PFC (vmPFC), and lateral OFC (lOFC; Kamigaki, 2019). Notably, it is clear now that mPFC is closely linked to fear-related behaviors in rodents, albeit with divergent functions of distinct subregions (Amodio and Frith, 2006).

The prelimbic (PrL) PFC neurons have been believed to encode sustained fear response in classic auditory fear conditioning (Burgos-Robles et al., 2009). It has long been demonstrated by *in vivo* unit recording that neurons in the amygdala elicit potentiated tone responses that correlate with the acquisition of conditioned fear (Quirk et al., 1995; Paré and Collins, 2000), while these neuronal activities last only a few hundred milliseconds and cannot be responsible for sustained fear responses in the auditory fear conditioning paradigm that last tens of seconds, suggesting the long-lasting fear responses should be stored in other brain structures. Using multichannel electrophysiological recordings in behaving rats, Burgos-Robles et al. (2009) revealed that sustained hyperactivity of the PrL neurons in response to the conditioned tone is correlated with freezing behavior suggesting that PrL neurons integrate inputs from the amygdala and other brain structures that form a top-down control of fear (Etkin et al., 2011) and contribute to the sustained fear expression. In support of this hypothesis, Karalis et al. (2016) found that the freezing response elicited by conditioned tone temporally coincided with sustained synchrony of 4-Hz oscillations in prefrontal-amygdala circuits. Contrary to the PrL, substantial evidence indicates that infralimbic (IL) sub-divisions of the mPFC is necessary for the extinction of conditioned fear (Quirk et al., 2006; Wang et al., 2018). Besides, collective evidence supports that OFC, another sub-division of the mPFC, also plays a crucial role in the regulation of conditioned fear (Sarlitto et al., 2018) and fear extinction (Rodriguez-Romaguera et al., 2015; Chang et al., 2018; Hsieh and Chang, 2020). However, in contrast to IL, activation of OFC negatively impaired extinction outcome (Rodriguez-Romaguera et al., 2015; Chang et al., 2018; Hsieh and Chang, 2020).

Despite a large amount of evidence supporting the essential function of PFC in conditioned auditory fear, its role in social fear is much less understood. By c-fos staining, our study found that after exposure to a conspecific mouse, the number of c-fos positive cells was increased in PrL but not in IL of mice with conditioned social fear, indicating a tight link between PrL neuronal activity and social fear expression (Xu et al., 2019). Further, pharmacological inhibition of mPFC with GABAa receptor agonist muscimol dramatically reduces social avoidance in mice with social fear elicited by either SFC or social defeat. As a high-order cerebral cortex, mPFC influences sociability by its projection to several brain areas, including the amygdala, hippocampus, and brainstem (Goodson, 2005). Interestingly, social defeat weakens neural functional connectivity between mPFC and periaqueductal gray (PAG), and selective chemogenetic inhibition of mPFC-PAG projection increases social avoidance (Franklin et al., 2017). Moreover, it has been clarified that layer 5 mPFC projection neurons inhibit excitatory inputs to glutamatergic neurons in PAG *via* presynaptic neuromodulatory mechanisms, and selective inhibition of these PAG neurons reduces social avoidance (Franklin et al., 2017). These observations provide mechanistic insight regarding the prefrontal modulation of social fear by a specific prefrontal projection to PAG.

## Cortical INs and Microcircuit

In the adult neocortex, the complex circuitry functions rely on a delicate balance between excitation and inhibition (Xu et al., 2013, 2019). Although neocortical INs represent a minority of total cortical neurons (10–20% in rodents; Kamigaki, 2019; Xu et al., 2019), they exhibit remarkable diversity in morphology, physiological properties, immunohistochemical characteristics, and connectivity (Kawaguchi and Kubota, 1997; Rudy et al., 2011; Xu et al., 2013; Hattori et al., 2017). Recent evidence suggests that neocortical INs can be divided into non-overlapping subgroups that expressing three different biomarkers: parvalbumin (PV, account for ~40% of total INs), the neuropeptide somatostatin (SST, account for ~30% of total INs), and the ionotropic serotonin receptor 5HT3a (5HT3aR, account for ~30% of total Ins; Rudy et al., 2011; Tremblay et al., 2016). Within 5HT3aR-expressing INs, ~40% of neurons also expressing vasoactive intestinal peptide (VIP), and the remaining are non-VIP INs (Rudy et al., 2011; Tremblay et al., 2016), which are the third-largest subtype of INs in the neocortex. In addition to PV, SST, and VIP, other biomarkers are often used to label cortical INs, including neuropeptides cholecystokinin (CCK), neuropeptide Y (NPY), and calcium-binding proteins calbindin (CB). However, these markers are expressed in overlapping populations of INs (Tremblay et al., 2016). The heterogeneity of INs is believed to facilitate their ability to perform complex operations.

INs actively gate information flow and sculpt network dynamics in a subtype-specific manner. PV^+^ and SST^+^ INs mainly target the perisomatic and distal dendritic regions of postsynaptic excitatory neurons, respectively (Hattori et al., 2017). By contrast to PV^+^ and SST^+^ INs, VIP^+^ INs mostly disinhibit excitatory neurons through inhibition of PV^+^ and SST^+^ INs (Tremblay et al., 2016). The PV INs can be further divided into fast-spiking (FS) basket and chandelier cells according to their morphology. Chandelier cells, also known as axo-axonic neurons due to their synaptic terminals specifically target the axon initial segment of PNs. In contrast, basket cells mostly target the soma and proximal dendrites of PNs and other INs. SST^+^ INs also constitute a diverse group and can be divided into Martinotti and non-Martinotti cells based on their different morphology (Tremblay et al., 2016). In the somatosensory cortex, Martinotti cells are mostly located in superficial (layer 2/3, L2/3) and deep (L5/6) layers, while non-Martinotti cells are mainly located in L4. Intriguingly, these two subtypes of SST^+^ INs also differ in terms of connectivity. In comparison to L2/3 Martinotti cells that predominantly target PNs, L4 non-Martinotti cells predominantly target local PV^+^ INs and disinhibit PNs (Xu et al., 2013).

Due to their distinct membrane properties and subcellular targeting on postsynaptic cells, it is suggested that distinct subtypes of INs contribute differentially to different cortical rhythmic oscillations. PV^+^ INs have fast kinetics of membrane property and inhibit local PNs at short latency. Also, PV^+^ INs target soma and perisomatic compartments of PNs which are essential subcellular regions to generate spikes, and therefore control the spiking output of PNs (Abbas et al., 2018). The nearby neural assemblies fire co-occurring spikes during the intervals of PV firing and follow the cycle of PV^+^ INs’ inhibitory inputs, which in turn leads to coherent oscillation in the local network with a high-frequency band (i.e., gamma oscillation; Cardin et al., 2009; Kamigaki, 2019). In contrast, SST^+^ INs have slow kinetics of membrane property and target distal dendrites, which can summate and integrate excitatory inputs of postsynaptic cells over a long time scale (Kamigaki, 2019), and maybe suitable for controlling long-range synchrony between neocortex and the sub-cortical or cortical afferents (Abbas et al., 2018). Although synchronized oscillations, particularly in the gamma band, are thought to facilitate information transfer within and across brain areas, their underlying mechanisms, as well as exact roles, remain a matter of debate (Veit et al., 2017). For example, Chen et al. (2017) showed that suppression of SST^+^ INs reduces both the spontaneous and visually induced enhancement of low-frequency band (beta) oscillation in the primary visual cortex (V1). In contrast, suppression of PV^+^ INs reduces oscillations in a broad frequency range (beta and gamma), suggesting that although PV^+^ INs are thought to generate cortical gamma oscillation (Cardin et al., 2009), they also strongly modulate low-frequency band activity. Consistently, another study conducted by Veit et al. (2017) also demonstrated that context-dependent visually induced low-gamma activity in the V1 also requires SST^+^ INs.

## Optogenetics and Chemogenetics Highlight Cell-Type Specific Role of INs in Social Fear

Although subtypes of cortical INs based on the expression of a single molecular marker may oversimplify the diversity of neural network organization (Kamigaki, 2019), this classification provides important opportunities to dissect cell-type-specific functions by recent innovative genetic tools. Optogenetics and chemogenetics are two of the most frequently used genetic techniques to specifically manipulate neuronal activity (Biselli et al., 2019). Optogenetics uses light-sensitive ion channels expressed in targeted cells allowing for neuronal depolarization or hyperpolarization with light illumination (Boyden et al., 2005), while chemogenetics uses designer receptors exclusively activated by designer drugs (DREADDs) expressed in targeted cells (Armbruster et al., 2007). The stimulatory DREADD hM3Dq (a modified human M3 muscarinic receptor) and the Gi-coupled hM4Di DREADD (a modified human M4 muscarinic receptor) have low affinity for the native ligand acetylcholine, but a high affinity for the synthetic ligand clozapine-N-oxide (CNO). Intraperitoneally or intracranial CNO administration causes a downstream signaling cascade leading to either increased firing (for hM3Dq) or silencing (for hM4Di) of the targeted neurons, allowing for prolonged neuronal excitation or inhibition. Although CNO has been widely used as a ligand to activate muscarinic-based DREADDs, sluggish kinetics and metabolic liabilities have also existed. Notedly, a new high-affinity and selective agonist deschloroclozapine (DCZ) can also combine muscarinic-based DREADDs with utility in both mice and nonhuman primates for a variety of applications (Nagai et al., 2020). Except for muscarinic-based DREADD, other types of DREADDs were also developed for chemogenetic manipulations. For example, kappa opioid receptor (KOR)-based DREADD is activated by Salvinorin B (SalB) allowing for inhibition of neuronal activity. Thus, co-expression of KOR- and M3-DREADDs allows remotely bidirectional modulation of activities of the same set of neurons with different ligands (Vardy et al., 2015).

Advances in tools for modulating or monitoring neuronal activity with cell-type specificity have expanded our understanding of the role of prefrontal INs in regulating social behaviors and dysfunctions. The development of genetically encoded calcium indicators, such as GCaMP (Chen et al., 2013), allows researchers to detect calcium transient in an individual neuron or a population of neurons (Ferguson and Gao, 2018). For example, by using fiber photometry to detect the overall activity of a distinct neuronal population, Selimbeyoglu et al. (2017) revealed that the activity of mPFC PV^+^ INs is increased in wild-type mice during social interactions with a conspecific compared to interactions with a novel object; however, in a genetic mouse model of autism, this difference was disappeared. Furthermore, either optogenetically increasing the activity of PV^+^ INs or decreasing the activity of excitatory PNs in the mPFC rescues social impairment in this autism mouse model (Yizhar et al., 2011). Together, these findings suggest that elevated prefrontal cellular balance of excitation and inhibition (E/I balance) causes a profound impairment in social behaviors, and that compensation of mPFC inhibition can rescue social deficits. Similarly, Courtin et al. (2014) demonstrated that fear expression in conditioned auditory fear is causally linked to the phasic inhibition of mPFC PV^+^ INs.

One extremely useful technique termed “optogenetic tagging,” which combines optogenetics with electrophysiological recording has been developed for *in vivo* identification of different neuronal subtypes at a single unit level (Zhao et al., 2011). This method is especially powerful for recording genetically identified subtypes of cortical GABAergic INs (Roux et al., 2014). Using this approach, we found that a majority of mPFC PV^+^ INs decrease their firing rate upon social confrontation in social fear-conditioned mice, whereas most PV^+^ INs maintain their activity in unconditioned mice (Xu et al., 2019). The activity of another two types of mPFC INs, SST^+^, and VIP^+^ INs was also monitored by fiber photometry in freely moving mice. It was found that the activity of SST^+^ INs is dramatically increased in social fear expression indicated by an increase in fluorescent signals when the mouse started each risk assessment behavior to approach a stimulus mouse. In contrast to SST^+^ INs, the activity of VIP^+^ INs is not altered during social fear expression (Xu et al., 2019). These observations clarified a significant association between the activities of distinct subtypes of mPFC INs with social fear expression. To further determine the causal relationship between mPFC INs activities and social fear expression, we employed a chemogenetic approach. After expression of hM3D on PV^+^ INs in the mPFC of social fear-conditioned mice, the social fear behavior is reduced upon CNO administration to activate those PV^+^ INs. Conversely, chemogenetic inactivation of mPFC SST^+^ INs also reduced social fear expression (Xu et al., 2019). These findings demonstrate that neuronal activities of dmPFC INs were potently modified by aversive social experience and that the hyperactivity of mPFC SST^+^ INs and hypoactivity of PV^+^ INs are critical mechanisms of social fear.

## SST^+^ INs Mediated Disinhibition in Social Fear

Besides feedforward inhibition and feedback inhibition, there is the third main type of “archetype circuit motifs” in the neural network, namely disinhibition (Letzkus et al., 2015; Tremblay et al., 2016; Möhler and Rudolph, 2017). Disinhibition is the removal of inhibition produced by one type of INs as a result of inhibitory action by another type of INs, and consequently enhances the activity of excitatory output neurons. It was firstly found in the hippocampus that a subpopulation of INs selectively innervates other GABAergic neurons, and this subpopulation includes calretinin-positive (CR^+^) INs (Gulyás et al., 1996) and VIP^+^ INs (Hajos et al., 1996). Such findings were then extended to the neocortex, where CR^+^ INs often preferentially target other CR^+^ INs and CB^+^ INs in L2/3 (Defelipe et al., 1999; Gonchar and Burkhalter, 1999; Caputi et al., 2009). Growing studies using slice recordings revealed that VIP^+^ INs have preferential connections with SST^+^ INs in diverse neocortices (Lee et al., 2013; Pfeffer et al., 2013; Pi et al., 2013). The VIP to SST disinhibitory connection is likely a general principle in the superficial layers of the neocortex (Tremblay et al., 2016). Furthermore, in L4 of the primary somatosensory cortex SST^+^ INs preferentially target PV^+^ INs, although their connection probability and synaptic strength are larger for PNs in the superficial layers (Xu et al., 2013). It seems that neocortical disinhibition of excitatory cells could be as powerful as direct inhibition (Tremblay et al., 2016).

**Figure 2:**
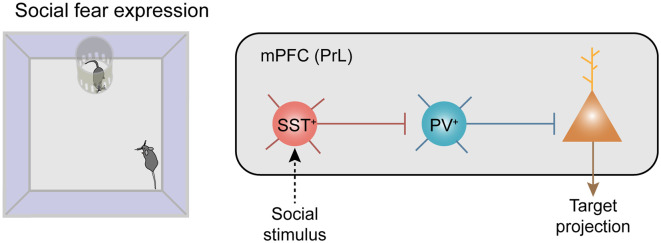
Recruitment of neocortical disinhibitory microcircuits in fear-related behaviors. Disinhibitory connectivity in the medial prefrontal cortex (mPFC) during social fear expression. Left: the behavioral paradigm of the social fear expression. Right: social stimuli recruit SST^+^ inhibitory interneurons (INs) which strongly inhibit PV^+^ INs and trigger disinhibition of the projecting principal neurons (PNs) in the mPFC.

All of the above observations were demonstrated in brain slices, it is critical to uncover whether this disinhibitory circuit operates *in vivo*, in particular under behavioral conditions. Here, we summarized well-known disinhibitory circuits in the literature, in particular for those with determined behavioral outcomes (Table 1). Recently, a couple of studies demonstrated that prefrontal SST^+^ INs mediated disinhibition also plays a critical role in the control of fear-related behaviors in rodents. In one of our recent studies, three lines of observations support that it is SST^+^ INs that inhibited PV^+^ INs during social fear expression (Figure 2; Xu et al., 2019). First, there was a robust enhancement of the neuronal activity of SST^+^ INs when the conditioned mouse approached the stimulus mouse, meanwhile, the activity of PV^+^ INs was largely suppressed. Second, after chemogenetical inactivation of SST^+^ INs, the activity reduction of PV^+^ INs was significantly suppressed during the social approach. Third, the inactivation of SST^+^ INs also decreased social fear behaviors in conditioned mice.

**Table 1:** Disinhibitory circuits and their physiological functions.

Disinhibitory circuit	Brain region	Physiological function	Reference
L1 INs-L2/3 VIP- PNs	V1	Sharpening orientation by sound	Ibrahim et al. (2016)
VIP-SST-PNs	V1	Enhancement of visual response by locomotion/top-down modulation	Fu et al. (2014) and Zhang et al. (2014)
CR-L2/3 CR/CB-PNs	V1/neocortex	NA	Defelipe et al. (1999), Gonchar and Burkhalter (1999) and Caputi et al. (2009)
VIP-SST-PNs; SST-PV-PNs; VIP-PV-PNs	Visual cortex	NA	Pfeffer et al. (2013) and Karnani et al. (2016)
VIP-SST-PNs	Auditory cortex	Auditory discrimination	Pi et al. (2013)
L1 INs-L2/3 PV-PNs	Auditory cortex	Auditory associative fear learning	Letzkus et al. (2011), Letzkus et al. (2015)
VIP-SST-PNs	S1	Enhancement of sensory processing by motor activity	Lee et al. (2013)
VIP-SST-PNs	S1	Intracortical LTP	Williams and Holtmaat (2019)
VIP-PV-PNs	S1	NA	Dávid et al. (2007)
L4 SST-PV- PNs	S1	NA	Xu et al. (2013)
L4 INs-L2/3 PV-L2/3 PNs	S1	NA	Gainey et al. (2018)
SST-PV- PNs	mPFC	Fear-related behaviors	Xu et al. (2019) and Cummings and Clem (2020)
SST-PV- PNs	mPFC	Spatial working memory	Kim et al. (2016)
SST-PV- PNs	Piriform cortex	NA	Sturgill and Isaacson (2015) and Large et al. (2016)
VIP/CR-unknown-PNs	Hippocampal CA1	Spatial Learning	Pardi et al. (2019) and Turi et al. (2019)
VIP/CR-O/A INs-PNs	Hippocampal CA1	NA	Gulyás et al. (1996), Hajos et al. (1996), Chamberland and Topolnik (2012), Tyan et al. (2014) and Pelkey et al. (2017)
VIP-PV-PNs	Hippocampal CA3	Spatial learning; novel object recognition	Donato et al. (2013)
PV-SST-PNs	BLA	Fear learning	Wolff et al. (2014) and Letzkus et al. (2015)
VIP-PV/SST-PNs	BLA	Auditory associative fear learning	Krabbe et al. (2019)
CeL SST-CeL PKC-δ-CeM output neurons	CeA	Fear responses	Ciocchi et al. (2010), Haubensak et al. (2010) and Li et al. (2013)
SST-CRF-output neurons; CRF-SST-output neurons	CeA	Selection of active and passive fear responses	Fadok et al. (2017)
Unknown INs-granule cells-mitral cells	Olfactory bulb	Odor discrimination	Nunes and Kuner (2015)
NAcLat D1 MSNs-VTA INs-DA	VTA	Reward-related behavior	Yang et al. (2018)
CEA INs-vlPAG INs-vlPAG glutamatergic neurons	PAG	Motor response of freezing	Tovote et al. (2016)

*BLA, basolateral amygdala; CB, calcium-binding proteins calbindin; CeA, central amygdala; CeM, medial part of central amygdala; CeL, lateral part of central amygdala; CR, calretinin; CRF, corticotropin-releasing factor; D1, D1-type DA receptors; DA, dopamine; LTP, long term potentiation; MSNs, medium spiny neurons; NAcLat, nucleus accumbens lateral shell; O/A, hippocampal CA1 stratum oriens/alveus; PAG, periaqueductal gray region; PKC-δ, protein kinase C-δ; PNs, principle neurons; V1, primary visual cortex; S1, primary somatosensory barrel cortex; vlPAG, ventrolateral periaqueductal gray region; VTA, ventral tegmental area*.

Consistently, using the auditory fear conditioning paradigm, an elegant study conducted by Cummings and Clem (2020) revealed that synaptic transmission, as well as auditory cue-evoked activity of prefrontal SST+ INs, are potentiated following cued fear learning. Besides, adopting diverse transgenic mice to independently tag SST^+^ INs and PV^+^ INs, they also provide direct electrophysiological evidence to show SST^+^ INs-evoked disinhibition in brain slices. The ratio of SST^+^ INs elicited monosynaptic inhibition in PV^+^ INs vs. surrounding PNs is strikingly increased in foot shock paired mice compared with that in the unpaired controls (Cummings and Clem, 2020), suggesting that fear conditioning shifts SST^+^ INs to preferentially inhibit PV^+^ INs and thereby produces disinhibition of PNs. This SST^+^ INs-mediated disinhibition is also reflected in behavioral tests. For instance, concurrent optogenetic activation of SST^+^ INs and PV^+^ INs abolished the fear-promoting effect of SST^+^ INs (Cummings and Clem, 2020), implying that this potent disinhibitory control is important for fear expression. Additionally, it was previously found that in the auditory cortex a disinhibitory microcircuit mediated by L1 INs, which robustly inhibit L2/3 PV^+^ INs and produce disinhibition of projecting PNs, plays a critical role in auditory fear learning (Letzkus et al., 2015).

It is known that distinct GABAergic neuronal populations in the mPFC receive differential long-range inputs from subcortical regions (Sun et al., 2019). In particular, SST^+^ INs in the mPFC receive more cholinergic inputs compared with PV^+^ or VIP^+^ INs, implying that acetylcholine release may preferentially drive SST^+^ INs (Sun et al., 2019). Using channelrhodopsin-assisted patching in awake mice, Muñoz et al. (2017) revealed that cholinergic modulation of SST^+^ INs in the somatosensory cortex provides a major excitatory drive to these neurons during whisking. Interestingly, Letzkus et al. (2011) found that in the auditory cortex an aversive stimulus (i.e., a foot shock) strongly recruits cholinergic afferents from the basal forebrain. Taken together, it is likely that acetylcholine release during fear expression could potentially recruit the SST^+^ INs-mediated disinhibitory microcircuit to reinforce mPFC output to drive social fear expression (Xu et al., 2019).

It is also important to determine downstream targets of the disinhibitory circuit mediated by SST^+^ INs for top-down behavioral controls in social fear. Anatomically, the PNs in the mPFC send their axons to multiple cortical and subcortical brain regions that are involved in the regulation of fear expression. Besides PAG that has been shown in the regulation of social fear (Franklin et al., 2017), other downstream brain regions such as the amygdala (Ciocchi et al., 2010; Wolff et al., 2014), paraventricular nucleus of the thalamus (PVT; Do-Monte et al., 2015; Penzo et al., 2015) are also possible targets since they are well known for various forms of fear regulation. Indeed, by c-fos staining, Cummings and Clem (2020) found that following optogenetic activation of prefrontal SST^+^ INs at 24 h after fear conditioning, a couple of remote downstream targets are identified, including BLA, PVT, lateral habenula, ventrolateral PAG and dorsomedial hypothalamus, suggesting that these brain regions are probably involved in this fear recruitment of SST^+^ INs-mediated disinhibition. Although PNs in the neocortex compose major output projections, GABAergic projections from the neocortex to subcortical regions have also been characterized recently (Lee et al., 2014). It was found that a subpopulation of PV^+^ FS INs in the mPFC projects to the nucleus accumbens (NAc) which release GABA, and activation of this projection elicits avoidance behavior in a real-time place preference task, suggesting that this projection is involved in aversive signaling (Lee et al., 2014). However, this projection is not likely involved in the expression of conditioned social fear since the firing activities of PV^+^ INs are indeed suppressed but not enhanced during social fear expression. Nevertheless, the exact brain networks downstream of mPFC outputs in control of social fear are to be dissected in future studies.

## Targeting SST^+^ INs to Cure Social Fear

The aforementioned potent disinhibitory microcircuit in the mPFC opens a new possibility by targeting SST^+^ INs to alleviate social fear behaviors. A couple of studies demonstrated that manipulation of prefrontal SST^+^ INs can alter animals’ defensive behaviors to fear response. For instance, we showed that chemogenetic inhibition of dmPFC SST^+^ INs causes a direct reduction of social fear (Xu et al., 2019). Consistently, Cummings and Clem (2020) showed that optogenetic inhibition of SST^+^ INs in the dmPFC markedly reduces freezing in mice 24 h after cue-foot shock pairing. On the other hand, optogenetic activation of SST^+^ INs *de nova* increases freezing in the absence of auditory cues. These observations suggest that inactivation of SST^+^ INs in the dmPFC could serve as an effective treatment option to mitigate fear responses.

The majority of antipsychotic drugs applied in the clinic to treat neuropsychiatric disorders have side effects due to their nonspecific actions outside the targeted brain regions. Besides, electric deep brain stimulation (DBS) or transcranial magnetic stimulation lacks cell-type specificity. A better understanding of brain node and network connectivity as well as advanced approaches like optogenetics and chemogenetics that can specifically manipulate targeted neuronal circuits could therefore be useful and of value to optimize therapeutic outcomes, although the invasiveness of these approaches limits their application in human beings (Jiang et al., 2017). Hopefully, progress in engineering will allow a new strategy of optogenetics-based DBS (Ramirez-Zamora et al., 2019) to selectively inhibit SST^+^ INs in the dmPFC with a high spatiotemporal resolution for future therapeutic purposes to treat social fear. For instance, using a potent fast red-shifted opsin ChRmine neuronal activations could be achieved by direct photostimulation above the surface of the intact skull (Chen et al., 2020). To avoid cranial surgery for viral delivery, systemic viral delivery of ChRmine was achieved to target dorsal raphe serotonergic neurons using engineered AAV to cross the blood-brain barrier, and activation of these neurons by transcranial light can promote social preference in a three-chamber test (Chen et al., 2020). Hence, a surgery-free and temporally-precise control of specific neural populations in animals is already doable. Currently, non-invasive optogenetics for neural manipulation at a depth of centimeters is not available for stimulating deep brain regions in humans (Chen and McHugh, 2020). However, from a translational point of view, it is likely feasible to target prefrontal SST^+^ INs given the fact that the cerebral cortex is the outmost structure of our brain.

## Concluding Remarks and Perspective

Focused on mainly animal studies, we have reviewed recent research advances in social fear. We have presented evidence that both SFC and sub-chronic social defeat in mice can induce core behavioral symptoms of SAD without alterations in locomotion, general anxiety, and depressive-like behaviors. A cell-type-specific alteration in neuronal activities of mPFC neurons represents an important mechanism underlying social fear. Further, a potent disinhibitory control of surrounding PNs by prefrontal SST^+^ INs plays a causal role in gating social fear behavior. In the future, identification of upstream inputs to the mPFC and also the exact downstream targets of the mPFC will help to draw a more complete picture regarding the circuit mechanism underlying social fear.

Generally, social-behavioral decisions depend on the dynamic integration of sensory information and the animal’s internal states (for review, see Chen and Hong, 2018). Correspondingly, for social fear expression, animals need to constantly combine both spatial and temporal sensory information with high-order memory representations originally acquired during fear conditioning. Integrating all this information with constantly changing internal states, animals eventually make a final decision and display appropriate defensive behaviors. The exact contribution of SST^+^ INs and the SST^+^ INs-mediated disinhibitory circuitry in each of these processes is another important question to be addressed.

Current evidence suggests that prefrontal SST^+^ INs exert a potent disinhibitory control over PNs during fear-related behaviors that are not necessarily specific in the social domain. Interestingly, it is recently reported that prefrontal SST^+^ INs are involved in discriminating the affective states of conspecifics in mice (Scheggia et al., 2020). Therefore, it is still possible that there exists a subpopulation of SST^+^ INs and their network are somehow wired specifically for processing social related information due to their distinct sensory inputs. Future studies using *in vivo* two-photo calcium imaging or microendoscope will be helpful to address this issue.

## Author Contributions

JW, YT, L-HZ and HX made a direct contribution to the work and approved it for publication. All authors contributed to the article and approved the submitted version.

## Conflict of Interest

The authors declare that the research was conducted in the absence of any commercial or financial relationships that could be construed as a potential conflict of interest.
